# Micromachining of Alumina Using a High-Power Ultrashort-Pulsed Laser

**DOI:** 10.3390/ma15155328

**Published:** 2022-08-02

**Authors:** Stefan Rung, Niklas Häcker, Ralf Hellmann

**Affiliations:** Applied Laser and Photonics Group, University of Applied Sciences Aschaffenburg, Würzburger Straße 45, 63743 Aschaffenburg, Germany; s170434@th-ab.de (N.H.); ralf.hellmann@th-ab.de (R.H.)

**Keywords:** ultrashort pulse laser, high rate ablation, Alumina, ceramics, processing strategy

## Abstract

We report on a comprehensive study of laser ablation and micromachining of alumina using a high-power 1030 nm ultrashort-pulsed laser. By varying laser power up to 150 W, pulse duration between 900 fs and 10 ps, repetition rates between 200 kHz and 800 kHz), spatial pulse overlap between 70% and 80% and a layer-wise rotation of the scan direction, the ablation efficiency, ablation rate and surface roughness are determined and discussed with respect to an efficient and optimized process strategy. As a result, the combination of a high pulse repetition rate of 800 kHz and the longest evaluated pulse duration of 10 ps leads to the highest ablation efficiency of 0.76 mm${}^{3}$/(W*min). However, the highest ablation rate of up to 57 mm${}^{3}$/min is achieved at a smaller repetition rate of 200 kHz and the shortest evaluated pulse duration of 900 fs. The surface roughness is predominantly affected by the applied laser fluence. The application of a high repetition rate leads to a small surface roughness Ra below 2 μm even for the usage of 150 W laser power. By an interlayer rotation of the scan path, optimization of the ablation characteristics can be achieved, while an interlayer rotation of 90${}^{{}^{\circ}}$ leads to increasing the ablation rate, the application of a rotation angle of 11${}^{{}^{\circ}}$ minimizes the surface roughness. The evaluation by scanning electron microscopy shows the formation of thin melt films on the surface but also reveals a minimized heat affected zone for the in-depth modification. Overall, the results of this study pave the way for high-power ultrashort-pulsed lasers to efficient, high-quality micromachining of ceramics.

## 1. Introduction

Technical ceramics are today an essential building block for different products in various applications, such as medical, automotive and electronics. Materials, such as alumina (Al_2_O_3_), zirconia (ZrO_2_) and alumina nitride (AlN) provide outstanding mechanical, thermal, electrical and chemical properties for high technical demands [1,2]. The probably most well-known and employed oxide ceramic is alumina, combining high electrical insulation (10${}^{14}$–10${}^{16}\Omega$m), high mechanical strength (600 MPa), high corrosion and wear resistance, low density (<4 g/cm${}^{3}$) and operating temperatures above 1000 ${}^{{}^{\circ}}$C [3].

These features enable the application as substrates and heat sink for power electronics, e.g., for engine control or charging units for e-mobility, or heavy duty forming tools and wear protection. However, the processing of this brittle material is a challenge for conventional manufacturing processes, such as milling or electrical discharge machining (EDM) [4,5]. Limiting factors for a suitable production technology are the material removal rate, quality factors, such as cracking and the resulting surface roughness.

Laser-based material processing is capable to enable multifarious techniques for the machining of technical ceramics. In recent years, both continuous wave (cw) and pulsed laser systems have been deployed for cutting [6,7], drilling [8,9], ablation [10,11] and even welding [12] of oxide and non-oxide ceramics. Especially for micro structuring, methods based on ultrashort pulsed (usp) laser systems, emitting laser pulses in the regime of pico- to femtoseconds (10${}^{-12}$–10${}^{-15}$ s), have attracted considerable interest [13,14,15].

Due to the possibility of small spot diameters in combination with a reduced heat generation, a precise manufacturing of metals, semiconductors and dielectrics is achievable. The usage of ultrashort pulses is associated with strongly reduced thermal effects, generally referred to as “cold ablation”, which in turn enables high machining quality with minimized thermal stress [16]. However, commonly usp laser based processes face a general conflict between a small material removal rate (mm${}^{3}$/min) and high precision.

With upcoming high-power industrial grade pico- and femtosecond lasers, there is a growing demand for machining strategies to deploy laser powers over 100 W to overcome the limiting factor of the material removal rate with coincident high quality surfaces for potential applications, such as dental implants [17,18]. A recent study shows the possibility of high-power ultrashort pulse laser ablation of alumina with minimized thermal load in the heat affected zone, which paves the way for processing strategies using high-power laser sources towards industrial implementation [19].

In this investigation, the key figures of high-power laser ablation of alumina are comprehensively studied. A Yb:YAG laser is used to evaluate the influence of pulse duration, laser power, laser fluence, pulse repetition rate, spatial pulse-to-pulse overlap and scanning strategies on the material removal rate, ablation efficiency and the resulting surface roughness. The results yield potential parameter combinations to enable a concurrence of either a removal rate up to approx. 40 mm${}^{3}$/min with an excellent surface roughness Ra of 1.8 μm using a high repetition rate of 800 kHz or a more rough-machine regime using 200 kHz achieving up to 57 mm${}^{3}$/min with a roughness Ra of 5 μm.

## 2. Materials and Methods

In this study, alumina (Ceramtec, Marktredwitz, Germany) with a purity of 96%, specified grain size of 3 to 5 μm and an initial substrate thickness of 1 mm is used for laser ablation experiments. The grain structure is shown in Figure 7a and the initial surface roughness is determined using laser scanning microscope (LSM, VK-X210, Keyence, Ozaka, Japan) to Ra 1.5 μm.

**Figure 1:**
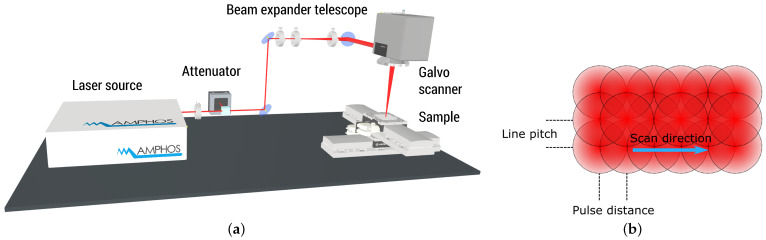
Schematic of (**a**) the laser setup for high-power micro machining and (**b**) pulse deposition.

For laser surface processing, we use a micromachining station (WSMH, Optec, Frameries, Belgium) equipped with a high-power ultrashort pulsed laser (Amphos 200, Amphos, Herzogenrath, Germany) having a tunable pulse duration between 900 fs and 10 ps (FWHM) and a repetition rate of up to 40 MHz. The maximum average power is 200 W with a maximum pulse energy of 1 mJ. For the presented ablation studies, the fundamental emission wavelength of 1030 nm is employed. Figure 1 shows the experimental setup for the surface treatment.

An external attenuator based on a rotating wave plate and a polarizer adjusts the energy of the laser. Using an adjustable zoom beam expander telescope, the raw beam diameter is accustomed to a diameter of 5.5 mm (1/e${}^{2}$).

A galvo scanner (Excelliscan 14, Scanlab, Puchheim, Germany) is used in combination with a telecentric lens (f = 163 mm) to focus the beam onto the sample with a spot diameter d${}_{f}$ of 50 μm (1/e${}^{2}$). All given values of the pulse energy E and the resulting laser fluence Φ are derived by the laser power P measured directly behind the processing optic. Thus, the parameter study extents up to 150 W average laser power impinging the specimen surface.

(1) $$\phi =\frac{2\;P_{avg}}{f_{r}\;d_{f}\;\pi }$$

**Figure 2:**
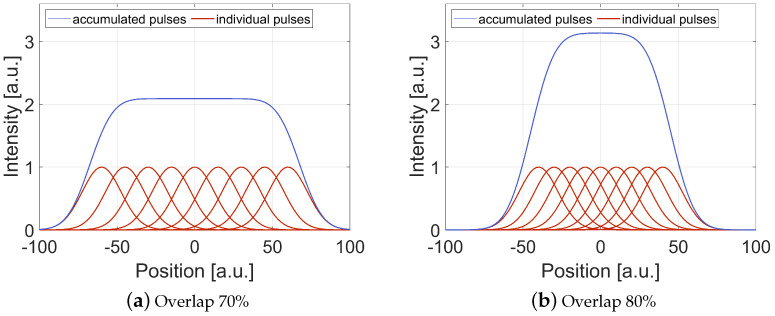
Accumulated intensity by equidistant placement of nine adjacent pulses with an overlap of (**a**) 70% and (**b**) 80%.

The pulse distance p between two adjacent pulses is chosen to be 15 μm for 70% overlap and 10 μm for 80% overlap, ensuring a homogeneous fluence distribution as well as a high scanning speed in both, scanning direction and perpendicular thereto (line pitch). This corresponds to scanning speeds vs. between 12,000 mm/s and 2000 mm/s with respect to the applied overlap and pulse repetition rate. With increasing pulse overlap, the accumulated intensity, i.e., the total number of pulses on a specific location, increases. Figure 2 depicts the accumulated intensity by equidistant placement of exemplarily 9 adjacent pulses with an overlap of 70% and 80% within a linear trajectory for the assumption of a Gaussian laser beam profile.

The integration of the adjacent beam profile over the number of pulses leads to the accumulated number of pulses in a specific location. The transfer from a 1D line to a 2D area can be achieved by the multiplication of the accumulated line number in the same way as described for the laser pulses. By the equidistant placement of two adjacent pulses and the line distance (c.f. Figure 1b) and the assumption of a Gaussian laser beam profile, the accumulated number of laser pulses N on a specific location can be calculated by

(2) $$N={\left(\int e^{-2\left(\frac{p^{2}\;n^{2}}{d_{f}^{2}}\right)}dn\right)}^{2}=\frac{\pi \;d_{f}^{2}\;er\;f{\left(\frac{\sqrt{2}pn}{d_{f}}\right)}^{2}}{8p^{2}}\approx \frac{\pi \;d_{f}^{2}}{8p^{2}}$$

where n describes the total number of laser pulses in one direction. If the desired ablation geometry is much larger than the spot diameter with respect to the required pulse distance, the value for the number of pulses can be assumed as high, leading to the simplified last argument in Equation (2). For an overlap of 70% N = 4.4 accumulated pulses whereas for 80% N = 9.8 accumulated pulses are absorbed by the location. In this study, a broad range of laser and processing parameters are under investigation, which are summarized in Table 1.

To evaluate the ablation characteristics, square cavities with an edge length of 1 mm are ablated using multifarious laser and processing parameters. The measurement of the resulted cavity depth d is performed using an optical 3D profilometer (VR 3200, Keyence, Ozaka, Japan). Each parameter combination is performed three times, and the values given in this study represent the mean value of the three individual results. Considering the cavity depth and the applied laser and processing parameters, the ablation rate $\dot{V}$(ablated volume per time) and the ablation efficiency *AE* (ablated volume per time and power) are calculated as followed.

(3) $$\dot{V}=\frac{d\;v\;p^{2}\;f_{r}}{n}$$

(4) $$AE=\frac{\dot{V}}{P_{avg}}$$

The optical inspection of the processed surfaces and the surface roughness measurement (Ra) are performed by a confocal laser scanning microscope. For a detailed visual inspection of the cavity surface we, additionally, use a scanning electron microscope (SEM, Maia3, Tescan, Brno, Czech Republic).

## 3. Results and Discussion

### 3.1. Laser Parameters

**Figure 3:**
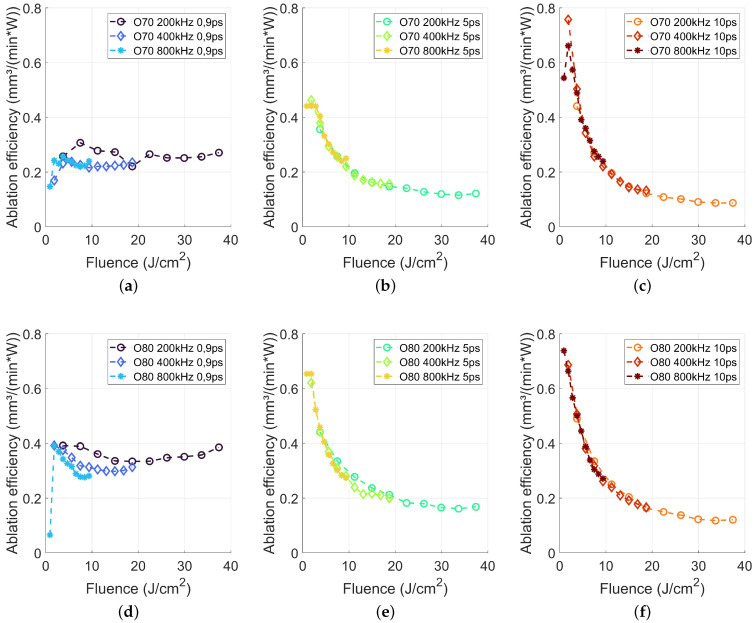
Ablation efficiency for 200, 400 and 800 kHz in dependence of the applied laser pulse length and laser fluence.

In a first step, we investigated the influence of laser fluence, pulse repetition rate, pulse duration and spatial pulse overlap. By a complete combination of these variable process parameters, 180 different parameter sets are studied to characterize the ablation behaviour for high-power laser ablation of Al_2_O_3_. The resulting cavity depths were used to calculate the ablation efficiency *AE* by using Equations (3) and (4) and summarized in Figure 3. For both overlap values, namely 70% and 80%, the ablation efficiency revealed a similar trend. Pulse durations in the picosecond regime showed high ablation efficiency for small laser fluences.

The highest *AE* of 0.7–0.76 mm${}^{3}$/(W*min) is reached using a fluence of 0.9–1.8 J/cm${}^{2}$ for a pulse duration of 10 ps for both, 70% and 80% pulse overlap. This is about a factor of three higher as reported by Beausoleil et al., reporting an *AE* below 0.23 mm${}^{3}$/(W*min) using 3 ps pulse duration, a repetition rate of 1.8 MHz and a spatial pulse overlap of 90% also for a laser wavelength of 1030 nm [20]. The main difference of our approach was the reduced pulse-to-pulse interaction, based on a lower repetition rate and a decreased spatial overlap, which was beneficial for the *AE*.

With increasing laser fluence, the *AE* decreased continuously, a typical behaviour for ultrashort laser ablation, being attributed to shielding effects using high laser fluences [21]. In addition, the *AE* remains about constant for increasing pulse repetition rates. As by increasing the pulse repetition rate from 200 kHz to 800 kHz, the average laser power is increased by the factor of four for identical laser fluence, and this suggests a minor impact of thermal effects onto the *AE*, even for laser powers up to 150 W.

With decreasing laser pulse duration to 5 ps, the maximum *AE* decreased for both investigated overlap values and all pulse repetition rates as compared to a pulse duration of 10 ps. A similar effect of decreasing maximum ablation efficiency with decreasing pulse duration is also shown for dielectric materials by Schwarz et al. [22] and specifically for alumina by Hodgson et al. [23]. However, the decrease of the *AE* with increasing laser fluence is less pronounced (c.f. Figure 3b,e) as compared to a pulse duration of 10 ps, while the decrease of the *AE* with increasing fluence remained unaffected by the pulse repetition rate.

The comparison between the pulse overlap of 70% and 80% revealed that increasing overlap led to a higher *AE*. Comparing pulse lengths of 10 and 5 ps shows, that for the application of high average laser powers it is more effective to use high repetition rates, i.e., a lower laser fluence, which enables a higher *AE*. From an application point of view, it is worthwhile to note that the increased pulse repetition rate requires a high scanning speed (c.f. Table 1), which is quite a challenge for current 2D galvo scanner in common optical configurations for laser micro machining.

A further diminution of the laser pulse duration to 900 fs revealed a different behaviour of the *AE* with respect to the laser fluence. For both overlap of 70% and 80%, an almost constant *AE* with increasing laser fluence is found. The pulse repetition rate of 200 kHz led to *AE* values between 0.25 and 0.28 mm${}^{3}$/(W*min) using 70% pulse overlap and between 0.34 and 0.39 mm${}^{3}$/(W*min) for 80% pulse overlap. The resulting *AE* for 400 kHz and 800 kHz was slightly below but also almost stable. The results from Figure 3a,d implied, that the application of a high laser fluence is very efficient using the here shortest available pulse duration of 0.9 ps, which led to a high material removal rate and thus to a higher productivity. These findings differ from the state of the art knowledge for processing of metals, ceramics and dielectrics [21,23,24].

**Figure 4:**
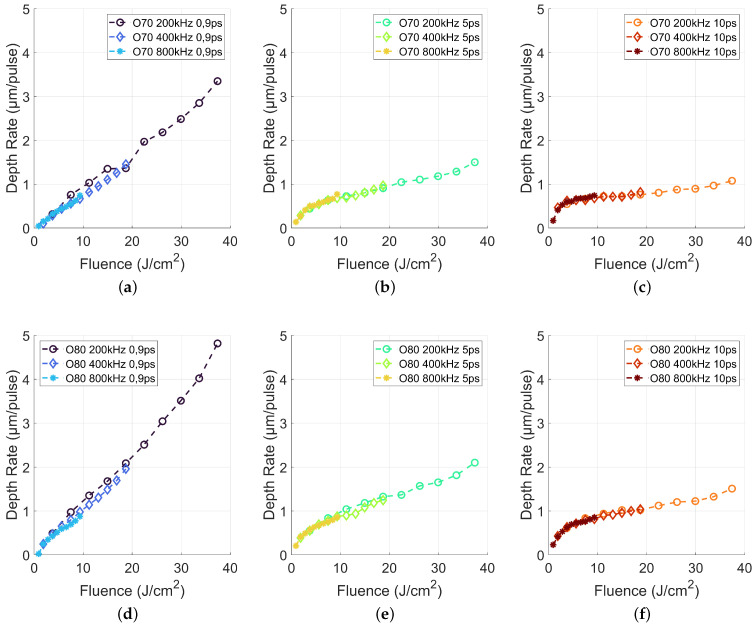
Depth rate for 200, 400 and 800 kHz in dependence of the applied laser pulse length and laser fluence.

Dividing the achieved cavity depth by the total number of absorbed pulses per area (accumulated number of laser pulses times number of layers), the depth rate, i.e., the cavity depth increase per laser pulse is calculated. Figure 4 depicts the depth rate against the applied laser fluence. The increasing slope of the depth rate with increasing spatial overlap and decreasing pulse duration underlines the previous given statements about the process efficiency.

An effective scalability of laser micromachining is possible, if a doubling of the applied laser fluence or laser power leads to a doubled depth rate, while the application of 5 and 10 ps leads to a smaller slope, laser micromaching using 900 fs revealed an almost 1-to-1 slope for 70% overlap and even slightly higher than 1-to-1 for an spatial overlap of 80%. However, high depth rates of up to 4.8 μm/pulse led to a rough surface. Thus, a further analysis of the generated surface roughness is required, which will be studied in the next step.

**Figure 5:**
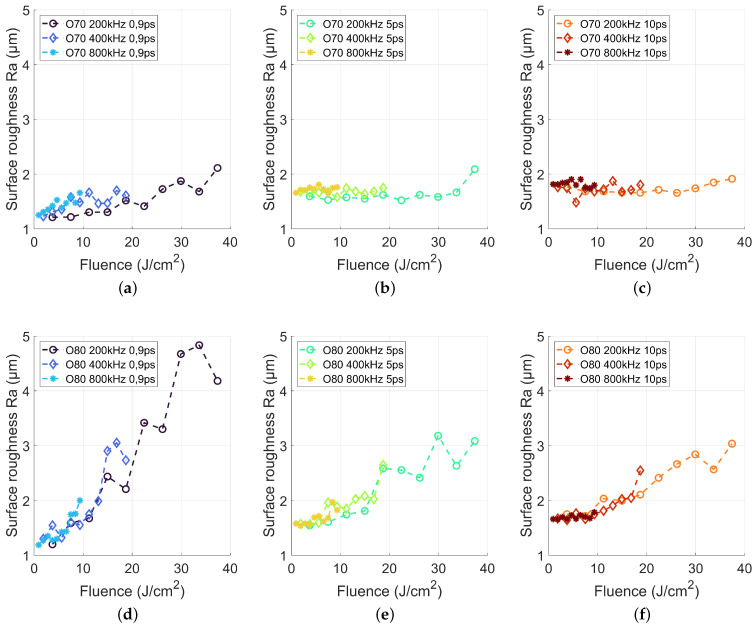
Surface roughness Ra for 200, 400 and 800 kHz in dependency of the applied laser pulse length and laser fluence.

Figure 5 summarizes the roughness Ra of the laser-processed surfaces for the 180 different parameter combinations. The overall finding was, that an overlap of 70% led to a small surface roughness regardless of the pulse duration, repetition rate and laser fluence, while an overlap of 80% led to a pronounced dependence of the surface roughness on fluence. For a pulse duration of 900 fs, a pulse overlap of 70% resulted in a slight increase of surface roughness with increasing laser fluence. However, the achieved roughness Ra was smaller than the initial roughness of the unprocessed surface (Ra = 1.5 μm) up to an applied laser fluence of about 19 J/cm${}^{2}$.

The SEM analysis of this low Ra regime showed a smooth melt layer on the ablated surface, which reduces the surface roughness. Zhang et al. [25] described this effect and attribute this to the recrystallization of nanoparticles excited by the ultrafast laser. The excited nanoparticles melted and recrystallized to form a layer of dense fine crystal structure on the ceramic surface, which resulted in the decreased roughness. With increasing pulse duration (Figure 5b,c) for a pulse overlap of 70%, the surface roughness is almost constant, ranging between 1.6 μm and 2 μm.

The SEM analysis (Figure 7) showed also a molten surface but with a larger amount of burr, which increased the surface roughness. In contrast, for a spatial pulse overlap of 80%, the surface roughness exhibits a pronounced dependence on laser fluence, while for a fluence below 10 J/cm${}^{2}$ a surface roughness below 2 μm is still achieved for all pulse durations. The roughness Ra increased with fluence for all pulse durations up to 5 μm for a pulse duration of 900 fs and about 3 μm for pulse durations of 5 and 10 ps, respectively, regardless of the employed repetition rate.

Comparing the depth rate from Figure 4 and the roughness in Figure 5 revealed a potential relation. With increasing depth rate, the roughness increased. A single pulse creates a depth of up to 4.8 μm and thus led to a rough surface. On the other hand, increasing laser fluence led to increasing melt pool depth even for ultrashort laser machining [26] and therefore to increased melt pool dynamics resulting in a higher surface roughness after the melt recast.

**Figure 6:**
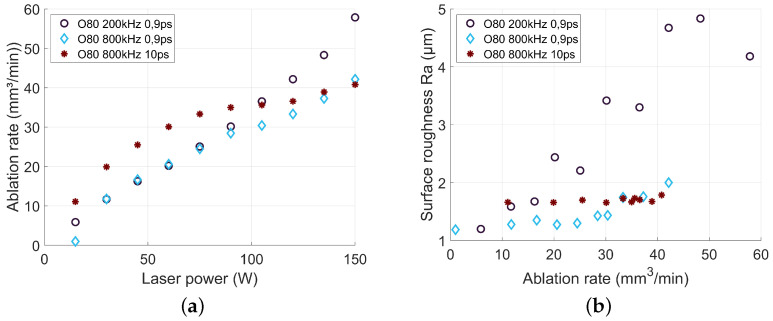
Ablation rate of Alumina as a function of the applied laser power (**a**) and resulting roughness values (**b**) for selected parameter combinations leading to a high rate ablation.

To transfer these findings into a highly productive and precise Al_2_O_3_ processing approach, also the ablation rate $\dot{V}$ has to be considered, as it is mainly responsible for the productivity. Figure 6 compares the achievable ablation rate versus applied average power and the correlation between resulting roughness and ablation rate for different combinations of pulse duration and repetition rate. Below an average laser power of 100 W, the high ablation efficiency achieved for a pulse duration of 10 ps (cf. Figure 3f) led to highest ablation rates using an overlap of 80% and a repetition rate of 800 kHz.

However, the decreasing ablation efficiency with increasing laser fluence limits the further scalability of the ablation rate to a maximum value of 41 mm${}^{3}$/min for 10 ps pulses. The almost constant ablation efficiency using a pulse duration of 900 fs (cf. Figure 3d), in turn, led to a nearly linear increase of the ablation rate with increasing laser power. Applying an average laser power of 150 W at a repetition rate of 200 kHz yields an ablation rate of 57 mm${}^{3}$/min. Figure 6b depicts the corresponding surface roughness as a function of the achieved ablation rate. Apparently, for 900 fs pulses the application of a smaller repetition rate, i.e., a high laser pulse energy, led to a distinct increase of the roughness Ra of up to 4.8 μm at higher ablation rates.

In turn, a laser processing strategy using a higher pulse repetition rate, here 800 kHz, enabled a high ablation rate with a constant surface roughness. For a pulse duration of 10 ps, the resulting surface roughness Ra was in range of 1.6–1.8 μm. By the application of 900 fs in combination with 800 kHz, it was possible to reduce the surface roughness to a range of 1.2 to 1.4 μm, which is on the level of the pristine material with ablation rates up to 30 mm${}^{3}$/min. This strategy makes it possible to combine a high rate laser ablation of Al_2_O_3_ with a high surface quality.

**Figure 7:**
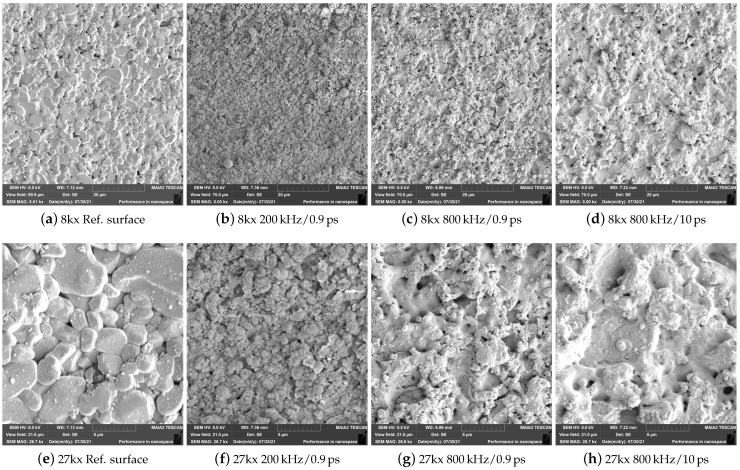
SEM analysis of unprocessed Al_2_O_3_ surface (**a**,**e**) and laser processed surfaces using an overlap of 80% and a high average power of 150 W in combination with (**b**,**f**) 200 kHz and 0.9 ps, (**c**,**g**) 800 kHz/0.9 ps and (**d**,**h**) 800 kHz/10 ps.

A detailed visual inspection of the laser-processed surfaces is shown in Figure 7, as determined by scanning electron microscopy. The upper row gives an overview using 8000× magnification whereas the lower row gives a more detailed view using 27,000× magnification. Figure 7a,e show the unprocessed surface with the typical grain structure of alumina for a further comparison. The remaining images show the results of selected machining strategies from Figure 6 for the highest applied laser power of 150 W. For all laser-processed areas, the grain-like structure was not visible anymore.

According to Leone et al. [27], the material removal mechanism was mainly attributed to direct melting and ablation of the ceramic as well as the vaporization-induced recoil force. With increasing laser fluence this recoil force led to hydrodynamic motion in the thin melt layer and therefore led to the increased roughness [28,29]. For 200 kHz and 900 fs, which showed the highest surface roughness of 4.2 μm, a specked distribution of small grain without a continuous surface area, i.e., no melting effect was visible on the surface.

By increasing the pulse repetition rate to 800 kHz, the continuous grain-like structure starts to vanish for a pulse duration of 900 fs and showed an even more molten character for a pulse duration of 10 ps. It is clearly visible, that the decrease of the peak intensity, either by an increase of the repetition rate or by elongated pulse duration, led to surface melting effects.

**Figure 8:**
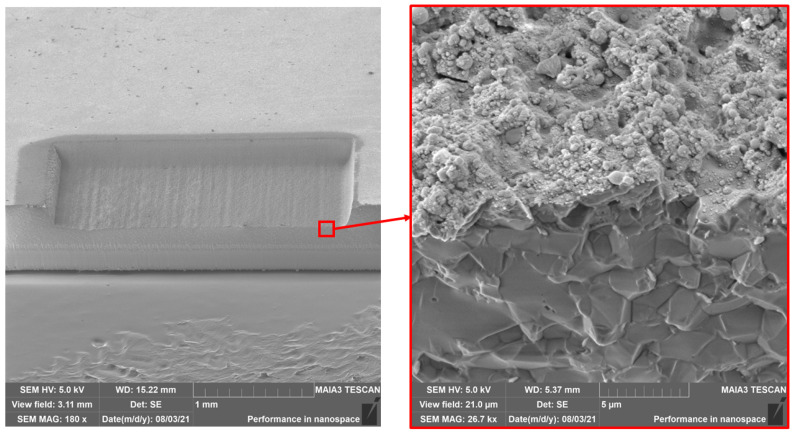
Cross section of an ablated cavity showing the surface melt effect of Al_2_O_3_.

The in-depth modification of alumina was analyzed by a cross section of the processed cavities. Therefore, a cavity was ablated using a laser power of 75 W, a pulse duration of 10 ps, a pulse repetition rate of 800 kHz, an overlap of 80% and with 7 passes. By backside scribing and cracking through the cavity, the in-depth modification of the ablated material was uncovered. Figure 8 depicts the 65${}^{{}^{\circ}}$ tilted overview and the detailed SEM image of this cross section.

The melting effect was clearly limited to the surface and the ablation process generates no visible in-depth modification. The sound grain structure of Al_2_O_3_ is directly under the melted surface, which was less than 0.5 μm thick. The inspection of the cross section revealed also, that the high-power laser processing using ultrashort laser pulses produced no cracks or other visual defects to the underlying material.

It is worthwhile to mention that a general increase of laser power can lead to a colorization effect, specifically a yellow cast of the processed surface that gradually extends into the surrounding untreated areas. Similar observations have been reported by Kodera et al. [30], Krell et al. [31] and Bärsch et al. [32] for wide band gap materials. It is suggested by Bärsch et al. [32] and Ackerl [33] to add an additional heat treatment to remove this colorization. Here, for Alumina, a post processing heat treatment, where the substrate was placed in an oven at 200 ${}^{{}^{\circ}}$C for 2 h, enabled a recovery of the initial optical appearance.

### 3.2. Scanning Strategy

Apart from the applied laser parameters, the scan path layout, which is defined by the direction of the laser feed and the angular change between ablated layers within a layer-wise ablation approach, also determines the machining results. The individual layer is defined by the desired geometry outline, which is filled with parallel laser scan tracks separated by the line pitch p. Two consecutive tracks can be either scanned in the same way, often referred to as linear mode, or the opposed direction, which is often refereed as a bi-linear mode.

On the one hand, the bi-linear mode can accelerate the processing speed due to the reduction of laser-off time by shorten the jump distance between two laser tracks. On the other hand, small deviations in the laser-on and laser-off delays at the start and the end of the laser track can lead to an undesired edge smoothing at the geometry outline. Our investigation showed, that the high-power ablation process was not affected by alternation of the laser track direction after each scanning path. Neither the roughness nor the ablation depth were significantly influenced. Using a spatial overlap of 80%, a repetition rate of 200 kHz, an average power of 80W, a laser pulse duration of 900 fs and 10 consecutive layers led to an average ablation depth of 216 μm and a roughness of 3.6 μm.

**Figure 9:**
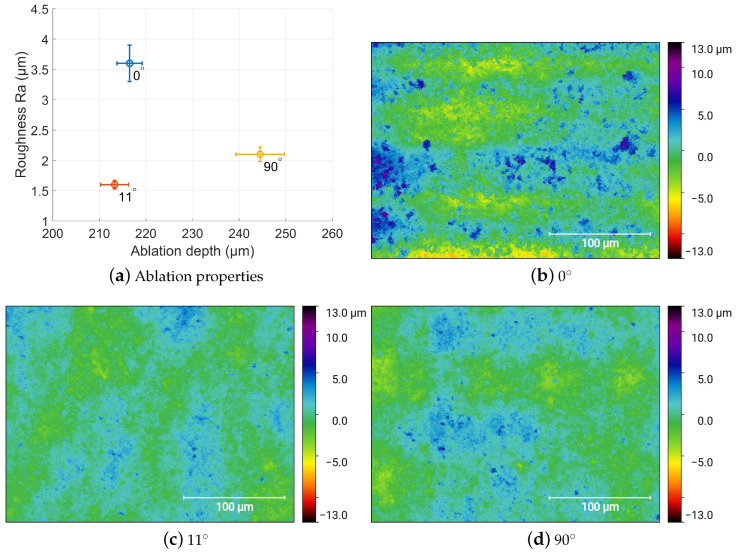
Influence of the interlayer rotation for 0${}^{{}^{\circ}}$, 11${}^{{}^{\circ}}$ and 90${}^{{}^{\circ}}$ on the achieved ablation depth and surface roughness. (**b**–**d**) Height map of the resulting surfaces.

The threefold execution combined with the linear and bi-linear scanning strategy showed no remarkable change. The influence of the direction and the manifold execution is represented by the error bars in Figure 9a. In addition, the slope of the cavity edge stayed unaffected with an angel of approx. 63${}^{{}^{\circ}}$. In addition to the linear and the bi-linear mode scanning approach, we additionally show the influence of a rotation between consecutive layers by a fixed angle.

Figure 9 shows the resulting ablation depth and the corresponding roughness for the interlayer rotation for 0${}^{{}^{\circ}}$, 11${}^{{}^{\circ}}$ and 90${}^{{}^{\circ}}$, while the rotation by 11${}^{{}^{\circ}}$ per layer had no influence on the ablation depth, the rotation by 90${}^{{}^{\circ}}$ led to a depth increase by approximately 13%. The influence of both, the orientation [34] and the order [35] of the laser scan path has been previously studied for several processes and materials, with a recommendation to rotate the angle between consecutive layers to improve process performance [36,37,38,39].

As soon as an interlayer rotation is applied, the resulted surface roughness decreased to 1.6 μm for 11${}^{{}^{\circ}}$ and 2.1 μm for 90${}^{{}^{\circ}}$. The comparison of the measured height map with standardized height scales in Figure 9b–d shows the surface modulation for the interlayer rotation for 0${}^{{}^{\circ}}$, 11${}^{{}^{\circ}}$ and 90${}^{{}^{\circ}}$. The height deviation was clearly visible for 0${}^{{}^{\circ}}$ rotation. A line-wise pattern appears parallel to the scanning direction; however, the periodicity is larger than the used line pitch of approx. 10 μm.

The least peak to valley deviation with respect to the mean level is produced using an interlayer rotation of 11${}^{{}^{\circ}}$, which also led to the smallest Ra value. This shows that a smooth surface can be achieved with a minimum amount of congruent laser scan tracks. However, the ablation depth can be maximized by using a perpendicular aligned line orientation.

## 4. Conclusions

We report on the laser micromachining of alumina using a high-power ultrashort pulsed laser with an average power up to 150 W. By varying the applied laser fluence, pulse duration, repetition rate and spatial overlap, we gain considerable insight into the process dependencies on these parameters with respect to efficiency and surface quality, in turn, allowing identifying preferential process strategies. In particular, the ablation efficiency is predominantly influenced by the applied laser fluence and laser pulse duration.

For the application of 5 and 10 ps pulses, the ablation efficiency drops with the increasing laser fluence. The maximum attainable ablation efficiencies are 0.7–0.76 mm${}^{3}$/(W*min), which were achieved using a laser fluence between 0.9 and 1.8 J/cm${}^{2}$ and a pulse duration of 10 ps. However, the application of a pulse duration of 0.9 ps led to an almost constant ablation efficiency up to 0.39 mm${}^{3}$/(W*min) even for a high laser fluence of 37 J/cm${}^{2}$. The pulse overlap shows, with respect to the achieved ablation depth, no significant influence on the process. Due to the required high scan velocity for smaller overlap values, a value of 80% led to a good compromise between the demands on the system performance and ablation quality.

With respect to the available laser power, we showed that a maximum ablation rate below 100 W can be achieved using a pulse duration of 10 ps and a repetition rate of 800 kHz. For higher laser powers, shorter pulses of 0.9 ps at a repetition rate of 200 kHz led to ablation rates up to 57 mm${}^{3}$/min. Laser processing using a high peak power, i.e., a high pulse energy and a short pulse duration, led to rough surfaces Ra up to 4.8 μm. SEM analysis further revealed that, in this regime, no extensive surface remelting was observed, which would smooth the surface. However, this surface melting layer is below a thickness of 0.5 μm, and no further depth modification is visible. A further decrease of the surface roughness can be achieved by a layer-wise rotation of the scan path using a small rotation angle of 11${}^{{}^{\circ}}$.

## Figures and Tables

**Table 1:** Overview of the laser and processing strategy parameters.

Constant			
Parameter	Symbol	Unit	Value/Range
Wavelength	λ	nm	1030
Beam quality	M${}^{2}$		1.3
Spot diameter	$d_{f}^{2}$	μm	50
**Variable**			
**Parameter**	**Symbol**	**Unit**	**Value/Range**
Average power	P${}_{avg}$	W	15–150
Pulse duration	τ	ps	0.9, 5, 10
Repetition rate	f${}_{r}$	kHz	200, 400, 800
Pulse energy	E${}_{P}$	μJ	19–750
Spatial pulse overlap	O	%	70, 80
Pulse distance = Line pitch	p	μm	15 (O 70%) 10 (O 80%)
Scanning speed	v	mm/s	3030 (O 70%,f${}_{r}$ 200 kHz) 6060 (O 70%,f${}_{r}$ 400 kHz) 12120 (O 70%,f${}_{r}$ 800 kHz) 2020 (O 80%,f${}_{r}$ 200 kHz) 4040 (O 80%,f${}_{r}$ 400 kHz) 8080 (O 80%,f${}_{r}$ 800 kHz)
Hatch rotation	α	${}^{{}^{\circ}}$	0, 11, 90
Number of scans	n		5, 7, 10

## Data Availability

Not applicable.
